# Attendance of psychosocial teen clubs and self-reported antiretroviral medication adherence: a cross section study of adolescents with perinatal HIV in the Kingdom of Lesotho

**DOI:** 10.3934/publichealth.2021044

**Published:** 2021-08-02

**Authors:** Sphiwe Madiba, Ntaoleng Mohlabane

**Affiliations:** Department of Public Health, School of Health Care Sciences, Sefako Makgatho Health Sciences University, Pretoria, South Africa

**Keywords:** adherence, adolescents, Kingdom of Lesotho, perinatal HIV, teen clubs, youth-friendly services

## Abstract

**Background:**

To address the problem of poor adherence among adolescents with perinatal HIV (PHIV), all clinics in Lesotho offer adolescent-friendly services and psychosocial support to improve their overall health outcomes and adherence. As a result, most adolescents with PHIV attend Teen Clubs as part of the package of youth-friendly HIV services. This study set out to determine whether attending Teen Clubs facilitates treatment adherence among adolescents with PHIV.

**Methods:**

In this cross-sectional study, data were collected from 130 adolescents aged 10–19 years who were aware of their HIV status and had attended three or more Teen Club sessions in selected clinics in rural district in Lesotho. Adherence was measured through self-report of last pills missed, based on the 7-days recall of pills taken. Descriptive statistics were used to analyse the data.

**Results:**

The median age of adolescents was 15 years, 56% were female, 37.7% were orphans, 41% were being cared for by their grandparents, 6.9% were living with siblings with no adult figure, and two were living on their own. The majority (93%) reported optimal adherence, 92% had not missed a clinic appointment in the past 30 days, and 74.4% knew that if they skipped doses, the viral load would increase and they would get sick. Over half (56%) had been reminded by their caregivers to take their medication and 96% talked to their caregivers regularly about their medication.

**Conclusion:**

A supportive environment provided through the Teen Clubs and in the home were the main facilitators for adherence. Strategies to improve adherence among adolescents should consider the importance of the involvement of caregivers in the adolescents' visits to their clinic.

## 1. Introduction

Young people, including adolescents represent a growing share of people living with HIV worldwide [1] due to the increased success of antiretroviral therapy (ART). The adolescent epidemic is a global epidemic, since the majority of children with perinatal HIV (PHIV) are growing into adolescence and young adults [2]. Lesotho, a small, landlocked country in sub-Saharan Africa (SSA) has an adult prevalence estimated at 25.6% for the 15 to 49 years age group, and 2.1% of children in the 0 to 14 years age group live with HIV [1],[3]. With the success of ART, there has been an increasing number of children with PHIV who are maturing into adolescents, thereby increasing the population of those who are chronically ill [4].

As ART access expands globally, adherence to a regimen of medication remains challenging [5]. Globally, adolescents with PHIV remain one of the population groups that has challenges in adhering to ART and their HIV-related complications continue to increase [6],[7]. Optimal adherence to ART is critical to the quality of life of children and adolescents with PHIV [8],[9]. Optimal ART adherence refers to taking medication as prescribed by the healthcare provider and attending clinic appointments and pharmacy refills as scheduled [10]. In the context of the universal test and treat strategy, ART adherence plays a key role in achieving the third “90”of the 90-90-90 strategy, which refers to the viral suppression of 90% of those on treatment [5]. Viral suppression is the ultimate goal and success of ART and is beneficial at both the individual and at the population level [11].

Adherence to ART is a strong determinant of the disease outcome. ART prevents AIDS, its sequelae, and death only if adherence is near perfect and treatment is long-term [2],[7]. However, levels of adherence are reported to vary in different studies and countries. Some countries report significantly higher adherence rates in paediatric populations, and others state that the high rates are due to the level of support the adolescent receives [12],[13]. Additionally, levels of adherence differ among adolescent age groups, and those 15 years and older have a higher risk of poor adherence than younger adolescents do [6].

Adherence is multifactorial, and the factors can be divided into those that hinder adherence and those that facilitate adherence. The factors include any combination of psychological, behavioral, environmental, and medication-related issues, which could explain why adolescents struggle with long-term adherence to ART [14]–[16]. Therefore, an understanding of the determinants of adherence is required in order to improve adherence and treatment outcomes [12]. Suboptimal adherence in adolescents is predicted by behavioral challenges such as peer pressure and concerns about body image [17]. Peer pressure and its effects are mostly felt in adolescence and can be the most important single determinant of adherence to PHIV. Adolescents with PHIV are also prone to treatment fatigue due to their having taken medication for a long time, sometimes since childhood, without ever seeing themselves as being sick [7]. Because ART requires near perfect adherence to be successful, adolescents with PHIV need support from their families, healthcare support, and psychosocial support that is given in terms of adherence sessions [4].

To address the problem of poor adherence among HIV-positive adolescents and improve their overall health outcomes, all clinics in Lesotho offer HIV and ART adolescent-friendly services on weekdays as well as on Saturdays. A key strategy to ensure the effectiveness of adolescent-friendly services and to address adherence and retention in care of children and adolescents with PHIV is through Teen Clubs. The Teen Clubs are significant in Lesotho where a sizeable proportion of adolescents are on the second or and third line of ART drug regimens because of poor adherence. These are monthly ART clinics for adolescents (10–19 years old) that provide clinical services and peer psychosocial support. The Teen Club package provides adolescents with dedicated weekend clinic time with sexual and reproductive health (SRH) education, disclosure support, peer mentorship, and a positive space for peer interaction through facilitated sports, arts and games, life skills, and access to SRH services [18].

Although the psychosocial Teen Clubs have been implemented in most clinics in Lesotho for more than four years [19], there are limited studies examining their effect on adherence. In countries where the Teen Club intervention has been introduced, a few studies have compared the retention rates between adolescents that were in a Teen Club and those in standard care [20],[21]. Data from a study conducted in Zimbabwe suggest that attending adolescents' specialised clinics improves adolescents' treatment outcomes. This study set out to determine whether attending psychosocial clubs facilitates ART adherence among adolescents with PHIV in Lesotho using self-reported accounts of adherence. Sub-optimal adherence is a major public health challenge, so understanding the adherence facilitators in this population could inform the design of interventions to improve adherence among HIV-positive adolescents [20].

## 2. Materials and methods

### 2.1. Study design

This cross-sectional study was conducted in nine adolescent-friendly clinics in the Mohale's hoek district of the Kingdom of Lesotho. The Kingdom of Lesotho is a landlocked country surrounded by South Africa. It is about 30,000 square kilometres in area and has an estimated population of 2.24 million. About 34% of the population live in urban areas and 66% in rural areas. Mohale's hoek is predominantly rural and mountainous, is located in the southern region of the Kingdom, and shares borders with South Africa. It has 15 health facilities, of which three are private clinics. The setting for the study was the 12 public facilities providing youth-friendly services, three of which were excluded from the study because they were hard to reach. The usual means of accessing them is via a helicopter or on horseback. The adolescent-friendly clinics provide services on weekdays and Saturdays for adolescents receiving ART. In addition, the clinics run Teen Clubs and offer a peer-support programme in which the facilitator is a trained healthcare worker such as a nurse, a counsellor, a social worker, or a peer educator. As already stated, the peer-support programme provides adolescents with counselling, addresses HIV knowledge, SRH education, adherence, disclosure, the acceptance of HIV status, and other psychosocial issues [18],[19].

### 2.2. Study population

The study participants were all children and adolescents aged between 10–19 years who were receiving medical services and ART from the selected facilities. Approximately 800 children and adolescents were enrolled in these facilities, according to the district clinic statistics at the time of the data collection. Of these, 130 adolescents with PHIV aged between 10 and 19 years were recruited using the convenience sampling method. The research team was restricted to recruit on the days the selected clinics offer youth friendly services, which is Fridays and Saturdays. The adolescents attended the Teen Clubs once a month, mostly on Saturdays to avoid missing out on school. Participants were eligible to participate in the study if they were aware of their HIV status and had attended three or more teen club sessions at the time of the data collection. Knowing their HIV status was a requirement for enrolment in the Teen Clubs. The nurse clinicians referred eligible participants to the research team after consultation, and all recruitment was done in the mornings. For adolescents who were unaccompanied to the clinic by a caregiver, the research team had to visit the clinic twice. On the first visit, eligible adolescents were identified and on the second visit, the interview was conducted if the caregiver has given written consent. The study excluded adolescents who were behaviorally infected, those who were not aware of their HIV status, and those who had not participated in the Teen Clubs. Those who satisfied the inclusion criteria were invited to participate in the study.

### 2.3. Data collection

A researcher-administered tool adapted from the AIDS Clinical Trials Group [22] was used to measure the level of adherence to ART. The questions were divided into three sections: (i) socio-demographic characteristics, (ii) clinical information, and (iii) adherence questions. Clinical information included questions such as the duration on ART, the ART start date, and the number of pills. Adherence was measured through self-report of the last pills missed, based on the 30 days and 7 days recall of the pills taken (validated through the medical records). Variables assessing the family's characteristics included the type of caregiver-child relation and orphan status. The tool was semi-structured, translated into Sesotho, and researcher-administered. The second author of this paper and a trained research assistant collected data on the days when the Teen Club sessions were offered. Training for the research assistant on the purpose of the study, the tool, and confidentiality was done prior to data collection.

The plan was to recruit only adolescents who were accompanied by caregivers for their routine appointments, but adolescents could come to the teen club sessions for ART refills unaccompanied. Since parental consent was required for those under the age of 18 years, the adolescents took information leaflets and consent forms home to obtain their caregivers' consent. Adolescents under the age of 18 years were interviewed only after obtaining caregivers written informed consent and their own written assent. The unavailability of caregivers to give consent in one clinic visit prolonged the data collection period to eight months since the research team had to visit clinics more than once to interview one participant. Written, informed consent was obtained from adolescents 18 years old and above. Informed consent and assent were administered in Sesotho. Face-to-face interviews with the participants were conducted in a private room in the absence of the parent/caregiver for those who had been accompanied to the clinic.

### 2.4. Data analysis

After data cleaning and coding, Stata version 13 was used for analysis. Descriptive statistics were used to summarise the socio-demographic characteristics, the clinical characteristics, and the levels of adherence of the participants. All categorical variables were reported as absolute numbers and percentages. The means of continuous variables like age and the duration on ART were computed.

### 2.5. Ethics approval and consent to participate

Ethical approval was sought from the Research Ethics Committee of Sefako Makgatho Health Sciences University [SMUREC/H/257/2017: PG] and permission from the Ministry of Health, Lesotho [REF: ID: 46-2018] and the District Health Management Team. To minimize the pressure on the adolescents to participate, the researchers explained to them and the caregivers that participation was voluntary and that the adolescents could withdraw from the study at any stage.

## 3. Results

### 3.1. Description of the participants

**Table 1.** Characteristics of the study participants (n = 130).

Variable	Categories	Freq.	Percent (%)
Gender	Male	56	43
	Female	74	57
Age category	10 years	3	2.3
	11–15 years	74	56.9
	16–19 years	53	40.8
	<15 years	77	59
	>15 years	53	41
Schooling	No	23	17.7
	Yes	107	82.3
Walked to the clinic	No	98	75.4
	Yes	49	24.6
Caregiver employment status	Employed	25	19.2
	Unemployed	100	76.9
	Pensioner	5	3.9
Living arrangements	Both parents	13	10
	Grandparents	26	26
	Mother	9	6.9
	Father	54	41.5
	Guardians	17	13.1
	Siblings	9	6.9
	Alone	2	1.5
Mother alive	Yes	81	62.3
	No	49	37.7
Father alive	Yes	53	41
	No	76	59
Accompanied to clinic	Yes	20	15.4
	No	110	84.6

The characteristics of the participants are described in Table 1. All the participants were at the clinic for their ART refills, 56% were female, 84.6% were not accompanied to the clinic by adult caregivers, and only 24.6% had walked to the clinic while the majority 75% used public transport to get to their nearest clinic, and 82.3% were currently in school. These who had used transport to the clinics had paid an average of R14.00 ($0.89) per trip. The median age of the participants was 15 years (range 10–19 years) with 41% above 15 years old. The participants were asked about their living arrangements in order to identify their main caregivers. Most (41.5%) of them were living with their grandparents, 6.9% were living with their siblings with no adult figure in charge, and two were living alone. Those who were being cared for by their grandparents included most of the orphans and those whose parents worked far from home. Over three quarters (77%) of the caregivers were unemployed. The participants were asked about their biological parents to determine those maternally orphaned, as well as paternal orphans. The majority (62.3%) reported their mother to be still alive, while the mothers of 37.7% were deceased. The opposite was the case with the fathers: the majority (58.5%) reported their fathers deceased. One quarter (24.6%) of the participants were classified as double orphans. They had lost both parents (Table 1).

### 3.2. ART-related history

The average number of tablets taken per day was two (range 1–9) and a significant proportion (n = 26, 20%) of the participants were taking more than three tablets per day and were on the second line and third line ART drug regimen. The majority (n = 50, 38.5%) of the participants had started taking ART between the ages of 11 and 15 years and the mean age of ART initiation was 9 years (SD = 5.8). Almost half (n = 63, 48.5%) had been receiving ART for not more than five years, the median time receiving ART was 4 years (SD = 4.19), 15.4% of the adolescents did not know when they had started ART, possibly because they had been receiving ART since birth.

**Table 2.** Adolescent's ART history.

Variable	Category	Frequency	Per cent
Age ART initiation	1–5 years	17	13.1
	6–10 years	26	20
	11–15 years	50	38.5
	16–19 years	17	13.1
	Don't know	20	15.4
Time on ART	<1 year	11	8.5
	1–5 years	63	48.5
	6–10 years	25	19.3
	11–15 years	11	8.5
	>15 years	3	2.3
	Don't know	17	13.1
Number of tablets	1 tablet	35	26.9
	2 tablets	48	36.9
	3 tables	21	16.2
	<3 tables	26	20

### 3.3. Self-reported adherence

Self-reported adherence was classified into optimal adherence, which correlates with missing zero to one dose on a seven-day missed dose recall (≥95%), non-adherence (less than 95%), or poor adherence, which correlates with missing two doses and more. The majority of the participating adolescents (83.1%, 108) reported no missed doses. Those who reported having missed doses within the seven days, 13 (10%) of them had missed one dose, 3 (2.3%) had missed two doses, 4 (3.1%) had missed three doses, and 2 (1.54%) had missed more than three doses. Those who missed doses cited lack of transport to collect ART (n = 3, 2.3%) and (n = 5, 3.6%) said they did not collect pills. The self-reported optimal adherence rate of the participants for the seven days recall was 93% and (6.9%) were non-adherent. The study did not find any association between non-adherence, gender, age, and duration on ART.

**Table 3.** Number missed ART doses in the past seven days.

	Frequency	Percent
Missed more than three days	2	1.54
Missed three days	4	3.1
Missed two days	3	2.3
Missed one day	13	10
Did not miss a dose	108	83.1
Optimal adherence	121	93.1
Non-adherence	9	6.9

**Figure 1.**
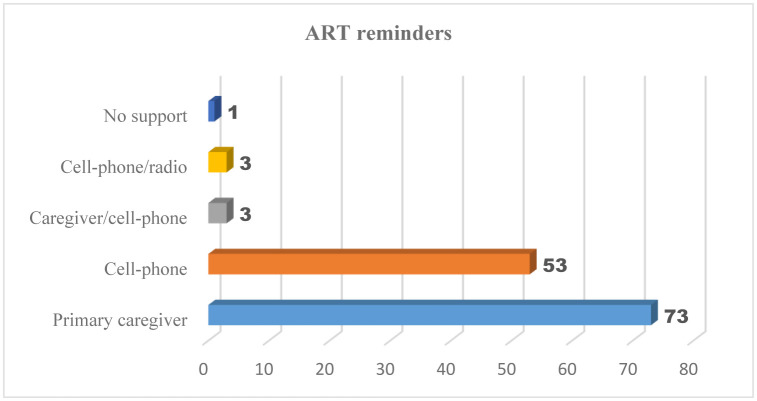
Types of reminders to take ART on time.

The majority (92.3%, 120) had not missed an ART clinic appointment in the past 30 days. Only 7.7% (10) reported to have missed an appointment, mainly due to lack of transport. Over half were reminded by their caregivers to take ART regularly and on time (n = 73, 56%), and over a third used cellphones as a reminder (n = 50, 38%) (Figure 1). Almost all (n = 125, 96.2%) talked to their caregivers about their medication.

### 3.4. Reasons for not skipping or stopping ART

All the participants said they would not stop taking ART or purposefully skip taking ART. The most commonly described consequence of skipping ART was getting sick (n = 93, 74.4%), and a small proportion said they would die (n = 16, 12.8%). Similarly, the main reason given for adhering to their ART regimen was that the medication keeps them alive (n=105, 81.5%).

**Table 4.** Self-reported reasons for adherence.

Reasons for not stopping to take ART	Frequency	Percent (%)
The medication is my life and keeps me alive	57	49,6
If I stop medication I will die	24	20.1
If I stop medication I will get sick	16	13.5
HIV and AIDS will multiply	20	16.8
Perceived outcomes of skipping ART doses		
HIV will increase and I will get sick	93	74.4
I will die	16	12.8
I will lose weight	8	6.4
CD4 will decrease	7	6.4

## 4. Discussion

This study assessed ART adherence among adolescents attending psychosocial Teen Clubs in rural Lesotho. The study found that 37.7% of the adolescents were maternal orphans, 41.5% were being cared for by their grandparents, and 77% of the caregivers were unemployed. The study showed that the mean age of enrolment in care was 15 years, and over half (51.6%) of the adolescents had been initiated on ART between the ages of 11 and 19 years. The findings are in keeping with those of other studies, both on the late diagnosis of HIV and late enrolment in care among the growing population of adolescents with PHIV [23],[24]. The reasons for the late initiation of ART in adolescents with PHIV, as noted in other studies, were fear, stigma, and waiting for clinical symptoms to appear [24]–[26]. The study further found that the mean duration on ART was four years, but three-quarters (70.1%) of the adolescents had been receiving ART for more than five years (range 5–15 years). A significant proportion (20%) of the participating adolescents were taking more than three tablets per day.

All the participants had been disclosed to fully and they were attending Teen Clubs at the time of the data collection. Although the current study did not collect data on the age when the participants received disclosure, research shows that full disclosure is often delayed until the child is above ten years old. In a study conducted in South Africa, Madiba and Mokgatle [26] found that disclosure occurred between 10 and 16 years of age. The mean age of initiation on ART in the current study suggests that the participants were disclosed to late in adolescence, possibly at the time when they were initiated on ART, between the ages of 11 and 15 years. Delayed disclosure has been associated with suboptimal adherence to treatment, poor health outcomes, virologic failure, and the need for more complex regimens [27],[28]. Clinical data from the study setting show that most adolescents are on the second line and third line regimen, following episodes of poor adherence. Hence, the attendance of Teen Clubs and the provision of adherence counselling has been adopted in Lesotho as standard care for children and adolescents with PHIV.

Good self-reported optimal adherence in the seven-day recall was reported by the participants, with 93% reporting optimal adherence. Concerning missing clinic appointments, 92% had not missed an appointment in the past 30 days. The results suggest that attendance of a psychosocial club is protective against non-adherence. The potential association between adherence and the attendance of psychosocial group sessions led by a professional facilitator had been documented [28]. The warm acceptance by and support of health care workers at treatment sites improve their adherence both to clinic visits and to their medical regimen [13]. It should be noted that a significant proportion (20%) of the participating adolescents were taking more than three tablets per day, up to nine tablets. Some of these adolescents could not benefit from the fixed dose formulations because they were switched to the second line and third line ART regimen due to poor virological outcomes before enrollment in the Teen Clubs. Recent data from Malawi suggest that exposure to Teen Clubs facilitates adherence, as these clinics serve as a resource for health education and psychosocial support for the adolescents [20]. The continuous information the HCWs give adolescents regarding the HIV illness and their medication facilitates adherence [13].

All the participants in the current study said that they would not intentionally stop taking their ART medication altogether. Half of them (49.6%) said they would not stop because ART keeps them alive, 20% said if they stopped taking medication they would die, 13.5% said that without their medication they would get sick, and 16.8% said that the prevalence of HIV and AIDS would increase. Research suggest that adhering to ART requires that the adolescents understand why they have to take ART, how it works, and how it benefits them [29]. Gross et al. [28] found that the comfort the adolescents feel in asking questions of the health provider and participating in group sessions was significantly associated with excellent adherence.

The study found that the adolescents had a comprehensive understanding of the negative outcomes of skipping doses of ART. Three quarters (74.4%) of them knew that if they skipped doses, their viral load would increase and they would get sick. A study conducted in Ghana reported similar findings [30]. Other studies have emphasised that adolescents' knowledge of the HIV disease is a strong determinant of adherence [31],[32]. In a study conducted in Botswana, Madiba and Josiah [16] found that comprehensive understanding of ART medication was a facilitator for adherence among adolescents with PHIV.

Although only 56% of the participants were being reminded by their caregivers to take their medication on time, other direct involvement of caregivers with the medication was observed. Majority (93%) had regular discussions with their caregivers about their medication. In this study, as in others, being reminded to take the medication on time, having regular discussions about the medication, and having the reason for taking the medication consistently explained were facilitators of adherence [16]. The role of social support especially from family members in influencing adherence outcomes has been reported elsewhere [7],[33]. The current study found that 84.6% of the participants had not been accompanied to the clinic by their adult caregivers, but having someone accompany them to the clinic and related good adherence is reported in other studies [16],[28]. The findings from this study and others underscore the need to include caregivers in interventions aiming at increasing ART adherence among adolescents with PHIV [33],[34]. Furthermore, the use of cell-phone reminders has been found to improve self-reported adherence among adolescents with PHIV in this study as well as in others [28],[30].

Our study employed self-report to measure ART adherence and being subject to social desirability, self-reports can overestimate adherence. Other methods that could be used to assess the level of adherence are viral load measures, but viral load monitoring in this population is poor due to attendance of Teen Clubs on weekends when laboratories and transportation is not available. Therefore, assessing short-term adherence provides a measure that could inform interventions to improve adherence among adolescents with PHIV in poor resourced settings and hard to reach populations such as Lesotho. Nevertheless, viral load measures remain important measures of optimal adherence. The study is further subject to selection bias because of the use of convenience sampling, the exclusion of adolescents for whom disclosure has not occurred, and exclusion of three hard-to-reach facilities. Adolescents in these facilities might have different experiences of the Teen Clubs. These render the findings unbefitting of being generalised to other populations of adolescents. The lack of comparison data is a limitation and there is a need for the performance of further studies that use a combination of measures of adherence among this population group using comparative data with adolescents who are not attending psychosocial Teen Clubs. Currently there is little consensus on what factors contribute most to ART adherence in children and adolescents in SSA.

## 5. Conclusions

A high proportion of the participants reported optimal adherence despite some of them being on complex drug regimens. The study found that a supportive environment provided through the Teen Clubs played a significant role in improving adherence. The participants had been enrolled in Teen Clubs as part of a package of adolescent-friendly services and psychosocial support to improve adherence following episodes of poor adherence. The participants had strong adherence, high knowledge of the importance of ART as well as family support.

Strategies to improve adherence among adolescents with PHIV should consider the importance of the involvement of caregivers in the adolescents' visits to their clinics. At the same time, the provision of professional-led Teen Clubs should be strengthened as a form of treatment support. In addition, future research should explore the adherence of those who do not attend the Teen Clubs to investigate the role of the caregivers in facilitating adherence in adolescents with a history of poor adherence.
